# The effects of training type and area size variations on the physiological and session rating of perceived exertion responses during male judo matches

**DOI:** 10.5114/biolsport.2024.127381

**Published:** 2023-05-25

**Authors:** Nizar Houcine, Ibrahim Ouergui, Anissa Bouassida, Emerson Franchini, Ezdine Bouhlel

**Affiliations:** 1 High Institute of Sport and Physical Education of Ksar Said, University of Manouba, Tunisia; 2 Research Unit: Sport Sciences, Health and Movement, UR22JS01, University of Jendouba, Kef 7100, Tunisia; 3 High Institute of Sport and Physical Education of Kef, University of Jendouba, Kef 7100, Tunisia; 4 Martial Arts and Combat Sports Research Group, School of Physical Education and Sport, University of São Paulo, São Paulo, Brazil; 5 Laboratoire de Physiologie de l’exercice et Physiopathologie, de L’intégré au Moléculaire “Biologie, Médecine, Santé”, UR12ES06, Faculty of Medicine Ibn El Jazzar, University of Sousse, Sousse 4000, Tunisia

**Keywords:** Combat sports, Physiology, Specific training, Martial arts, Physiology

## Abstract

Modified exercise prescription in judo is commonly used to activate the energy systems in different magnitudes. In order to study the physiological and rating of perceived exertion (RPE) responses according to area sizes (i.e., 4 m × 4 m, 6 m × 6 m and 8 m × 8 m) and training mode variations (i.e., groundwork, ne-waza; standing combat only, tachi-waza; and free combat, free randori), eighteen male judo athletes (age: 22.6 ± 1.8 years) were randomly assigned, on separate days, to 9 experimental conditions (3 area sizes × 3 training modes) with each condition lasting 4 min. Delta lactate [La] was calculated based on the blood lactate values measured before and after every condition. Heart rate (HR) was measured during and after each bout and RPE recorded at the end of each combat. The results showed that mean and peak HR, percentage of maximum HR (% HR_max_), delta [La] values and RPE scores were lower in 4 m × 4 m compared to 6 m × 6 m and 8 m × 8 m, and in groundwork training mode compared to standing combat and free randori (all p < 0.001). Furthermore, the 6 m × 6 m condition induced lower delta [La] values than 8 m × 8 m (p < 0.001) and free randori resulted in higher RPE scores than standing combat (p = 0.001). In conclusion, different training variables can be easily manipulated in a variety of different ways to specifically activate the energetic systems. Focusing on groundwork, the 6 m × 6 m area size was found to be the most suitable condition to induce a higher cardiovascular response, while the standing combat and free randori in 6 m × 6 m resulted in increased glycolytic activation compared to the groundwork condition.

## INTRODUCTION

Judo competition is physiologically demanding; therefore, a high level of aerobic and anaerobic fitness is required [1]. Exercise is prescribed in such a manner as to activate and thus train different physiological systems, thereby enhancing judo athletes’ abilities to cope with, prepare for and recover from training and competition [1]. In support of this, several previous studies in judo were conducted to improve training programmes aiming for competition success by developing the physical fitness as well as the technical-tactical skills [1, 2, 3]. It is relevant to note that physiological and perceptive responses were studied in various judo-specific exercises. It was reported that continuous uchi-komi exercises (i.e., technique repetition without throwing) induced low glycolytic demands while the intermittent protocols resulted in higher demands, suggesting therefore that the uchi-komi training modality may improve aerobic and anaerobic fitness [1, 2, 4]. When the technique repetition training modality was performed by throwing the partner (i.e., nage-komi), different physiological responses were recorded based on the technique used [1]. Specifically, when seoi-nage (i.e., an arm technique) was used during nage-komi, the oxidative demand was higher compared to o-uchi-gari (i.e., a leg technique) [5]. Regarding training mode, it has been previously shown that continuous randori (i.e., combat or fight practice; sparring) elicited higher cardiovascular strain compared to the intermittent randori [6]. Additionally, ne-waza sessions (i.e., groundwork combat) have been reported to be less demanding in terms of glycolytic activation (inferred from blood lactate concentration) compared with standing combat, which can be a more appropriate mode for aerobic fitness development for judo athletes [7].

Recently, Ouergui et al. [3] investigated the physiological responses and rating of perceived exertion (RPE) during different judo combat conditions in female judo athletes. They found that RPE scores were higher in 4 m × 4 m compared with 8 m × 8 m area size. The post-combat lactate [La] values were higher in 4 m × 4 m compared to 6 m × 6 m and 8 m × 8 m area size, and in free randori compared with the 3:1 ratio condition. However, no changes were reported for heart rate (HR) values. It is known that there are several differences regarding a judo match’s time-motion characteristics between male and female judo practitioners (e.g., male judo athletes spend longer time in standing combat than female judo athletes) [8], which may be related to hormonal [9], cardiovascular and anaerobic capacity [10], as well as technical-tactical [10, 11] aspects. Thus, based on these male-female differences [8, 9, 10, 11, 12], it seemed unlikely that the outcomes obtained with female judo athletes [3] would be replicated in male judo athletes. To the authors’ knowledge, no studies have investigated the physiological responses and perceived exertion in male judo combat when altering area size and varying the training mode.

Therefore, the aim of this study was to examine the effect of altering area sizes (4 m × 4 m, 6 m × 6 m and 8 m × 8 m) and training mode variation (groundwork, standing combat and free randori) on physiological (i.e., [La] and HR responses) and RPE responses in male judo athletes. It was hypothesized that physiological and perceptive responses would be higher in 4 m × 4 m compared to other area sizes [3], while lower responses would be attributed to groundwork in comparison to standing combat due to its high energetic demand (most of the throwing techniques used are with the maximum physical lever) [13]. When considering both area size and training mode, the main hypothesis was that 4 m × 4 m in standing combat would induce the largest responses.

## MATERIALS AND METHODS

### Participants

A priori power analysis was calculated using the G*Power software (Version 3.1.9.4, University of Kiel, Kiel, Germany) using the F test family (ANOVA: repeated measures, within-between interaction). The analysis revealed that a total sample size of 12 subjects would be sufficient to find significant differences (effect size f = 0.25, α = 0.05) with an actual power of 83.16%. Eighteen male judo athletes volunteered to participate in this study (mean ± SD, age: 22.6±1.8 years; height: 174.6±2.6 cm; body mass: 73.3±4.4 kg; and judo experience: 9.8 ± 1.5 years). All athletes were grouped according to their weight divisions [lightweight (-73 kg) and half-mid-dleweight (-81 kg) categories] and had participated regularly in judo tournaments for more than 2 years. They were also undertaking a similar training regime (3–5 times a week, 2 hours per session). The athletes did not present any medical restrictions during the experimental period and refrained from any strenuous exercises 48 hours before each experimental session. This study was conducted according to the Declaration of Helsinki for human experimentation and approved by a local research ethics committee. Written informed consent was obtained after a detailed explanation about the aims and risks involved in the investigation.

## PROCEDURES

### Study design

Based on variation in area size (4 m × 4 m, 6 m × 6 m and 8 m × 8 m) and training mode (groundwork, standing combat and free randori), 9 experimental conditions were randomly performed during a maximum period of 30 days with recovery duration of at least 48 h (but not more than 72 h) between 2 successive sessions [3]. Before each condition, the athletes were assigned to a standardized warm-up session (i.e., 15 minutes of jogging and dynamic stretching). The baseline measures were determined after 3 minutes of passive rest. The sessions were always conducted at the same time of the day (10 am to 12 pm) and took place at the training centre with daily controlled temperature (~20°C). All athletes competed against each other, were exposed to the same match duration and were instructed to continue the combat even when an ippon was scored [3]. The experimental protocol was directed by 2 investigators ensuring the athletes’ safety. One week before the beginning of the investigation, the judo athletes were familiarized with the tests and the randori sessions order. They accomplished the 20 multistage shuttle run test [14] to determine their maximal HR (HR_max_).

### Study measures

Blood samples were taken 10 min before and immediately after each condition from the fingertip, after which [La] was measured using the Lactate Pro2 Analyzer (Arkray, Tokyo, Japan). Blood lactate concentration at pre- and post-conditions was determined and delta lactate (Δ) was calculated for the main analyses. Heart rate was measured continuously with a 5-second interval using a telemetric system (Polar Team2 Pro System, Polar Electro OY, Kempele, Finland). A transmitter belt worn by each judo athlete communicated via Bluetooth to sideline software for display of multiple players’ HR. The Polar Pro sensor was handed out to all judo athletes before the start and was then collected at the end of the experimental conditions. After the completion of each condition, data were uploaded, and HR was analysed through Polar Flow (Polar Electro Oy, Kempele, Finland). For each data collection, the athletes wore the same transmitter belt to prevent recording differences. HRpre, mean (HRmean) and peak (HRpeak) values were used for the analysis. After being familiarized with the scale, athletes reported their RPE scores using a CR-10 scale [15] 30 min after each combat session.

### Statistical analysis

The statistical analysis was performed using SPSS 20.0 statistical software (SPSS Inc, Chicago, IL, USA). Univariate normality was checked and confirmed using the Kolmogorov-Smirnov test. Data were analysed using a two-way analysis of variance (area size [4 m × 4 m, 6 m × 6 m, and 8 m × 8 m], training mode [groundwork, standing combat and free randori]) with repeated measurements to compare HRmin, HRmean, HRpeak, %HR_max_, delta lactate and RPE. The sphericity was tested and confirmed using the Mauchly test. The Bonferroni test was used as a post-hoc procedure. Standardized effect size (Cohen’s d) analysis was used to interpret the magnitude of differences between variables and classified according to Hop-kins [16]: < 0.20 (trivial); 0.20–0.60 (small); 0.60–1.20 (moderate); 1.20–2.0 (large); 2.0–4.0 (very large); and > 4.0 (extremely large). Moreover, upper and lower 95% confidence intervals of the difference (95% CIds) were calculated for corresponding variation. The statistical significance level was set at p < 0.05.

## RESULTS

The mean value of HR_max_ recorded during the multistage 20-m shuttle run test was 199 ± 4 beats · min^−1^. Figure 2 presents heart rate, delta lactate concentration [La] and session rating of perceived exertion (RPE) responses to different experimental conditions resulting from the interaction between area size and training mode.

For HRmin values, there was no area size, no training mean and no interaction effects (p > 0.05).

**FIG. 1:**
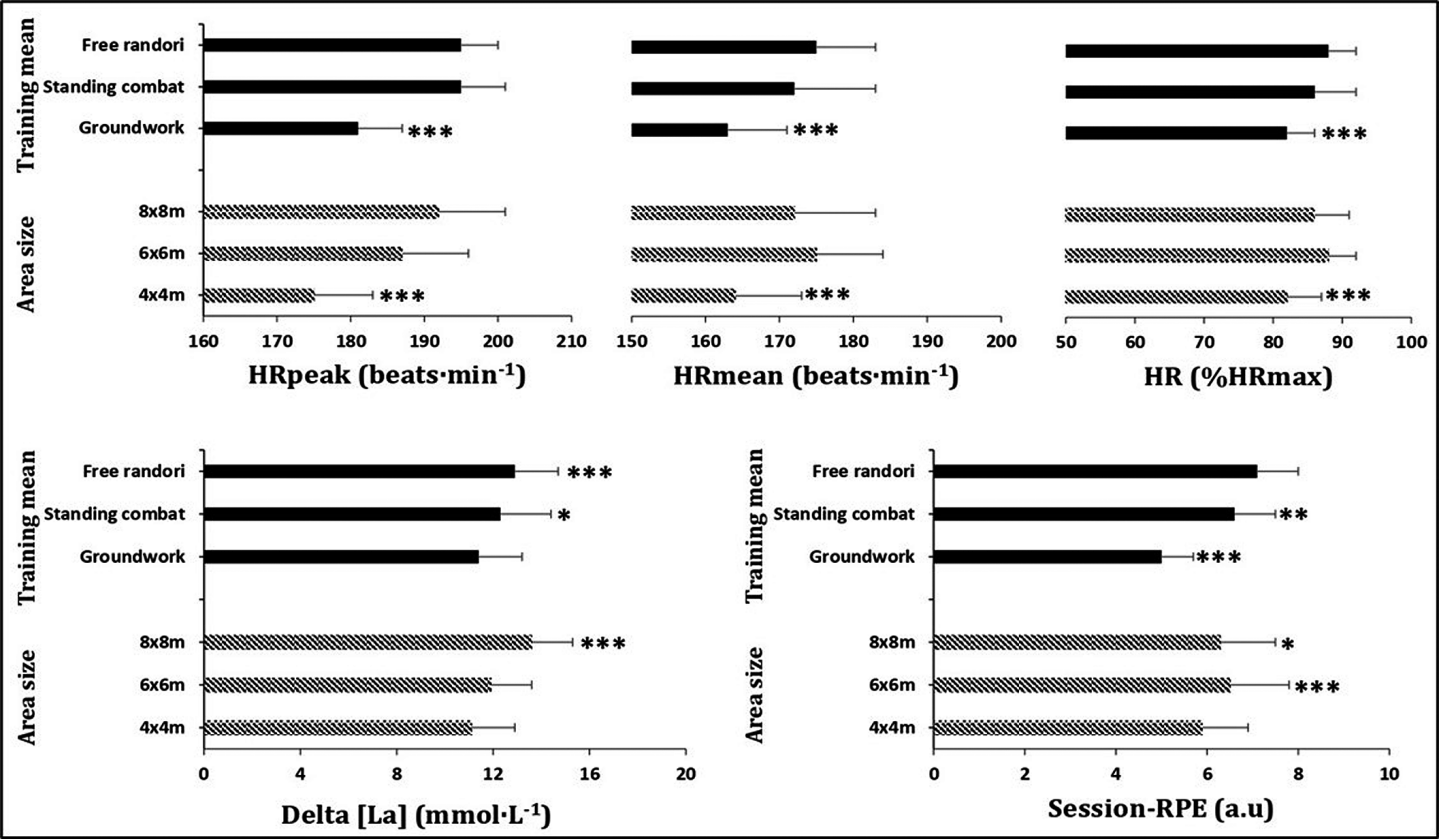
Area size and training mode main effects on heart rate, delta lactate concentration [La] and session rating of perceived exertion responses during different experimental conditions. * different at p < 0.05: Delta lactate was higher in standing combat compared to groundwork; Session RPE was higher in 8 m × 8 m in comparison to 4 m × 4 m. ** different at p < 0.01: Session RPE was lower in standing combat compared to free randori. *** different at p < 0.001: HRmean, HRpeak and %HR_max_ were lower in 4 m × 4 m compared to 6 m × 6 m and 8 m × 8 m; HRmean, HRpeak, %HR_max_ and session RPE were lower in groundwork compared to standing combat and free randori; session RPE was higher in 6 m × 6 m compared to 4 m × 4 m; delta lactate was higher in 8 m × 8 m in comparison to 4 m × 4 m and 6 m × 6 m; delta lactate was higher in free randori compared to groundwork. a.u. arbitrary unit; RPE: rating of perceived exertion; HRpeak: peak heart rate; HRmean: mean heart rate; %HR_max_: percentage of maximum heart rate.

For HRmean, HRpeak and %HRmax there was an area size effect (F_2,153_ = 29.769, F_2,153_ = 9.747 and F_2,153_ = 31.050, respectively; all p < 0.001), with 4 m × 4 m resulting in lower values than 6 m × 6 m and 8 m × 8 m (HRmean: 95%CId= -15;-7 and -12;-4; d=-1.24 and -0.81 (large and moderate); HRpeak: 95%CId= -6;-1 and -6;-1; d=-0.46 and -0.48 (moderate for both comparisons); %HRmax : 95%CId= -7;-4 and -6;-2; d=-1.28 and -0.81 (large and moderate); all p<0.001).. Moreover, a training mode main effect was detected for the same HR parameters (F_2,153_ = 34.048, F_2,153_ = 118.058 and F_2,153_ = 35.597, respectively; all p < 0.001), with groundwork eliciting lower values in comparison to free randori and standing combat (HRmean: 95%CId= -15;-8 and -12;-5; d=-1.42 and -0.87 (large and moderate); HRpeak: 95%CId= -16;-11 and -16;-11; d=-2.31 and -2,23 (very large for both comparisons); %HRmax: 95%CId= -8;-4 and -6;-3; d=-0.87 and -1.5 (moderate and large); all p<0.001). For delta lactate, there was an area size effect (F_2,153_ = 31.050; p < 0.001), with 4 m × 4 m producing lower values than 6 m × 6 m and 8 × 8 m ((95%CId= -2;-0.13 and -3;-2; d=-0.47 and -1.44 (moderate and large); p=0.29 and p<0.001, respectively) and 6 m × 6 m resulting in lower values than 8 m × 8 m (95%CId= -2;-1; d=-1.01 (moderate); p<0.001). Moreover, a training mode main effect was detected (F_2,153_ = 35.597; p < 0.001), with groundwork resulting in lower values in comparison to free randori and standing combat (95%CId= -2;-1 and -2;0; d=-0.83 and -0.4 (moderate and small); p<0.001 and =0.015, respectively). Finally, for session-RPE there was an area size effect (F_2,153_ = 9.574; p < 0.001), with 4 m × 4 m resulting in lower values than 6 m × 6 m and 8 m × 8 m (95%CId= -1;-0.16 and -1;-0.13; d=-0.51 and -0.37 (small for both comparisons); p<0.001 and p=0.014, respectively). Moreover, a training mode main effect was detected (F_2,153_ = 119.034; p < 0.001), with groundwork resulting in lower values in comparison to free randori and standing combat (95%CId=-2;-2 and -2;-1; d=-3.08 and -2.29 (very large for both comparisons); p<0.001, for both comparisons) and standing combat resulting in lower values compared to free randori (95%CId=-1;-0.16; d=-0.63 (moderate); p=0.001) (Figure 1).

**FIG. 2:**
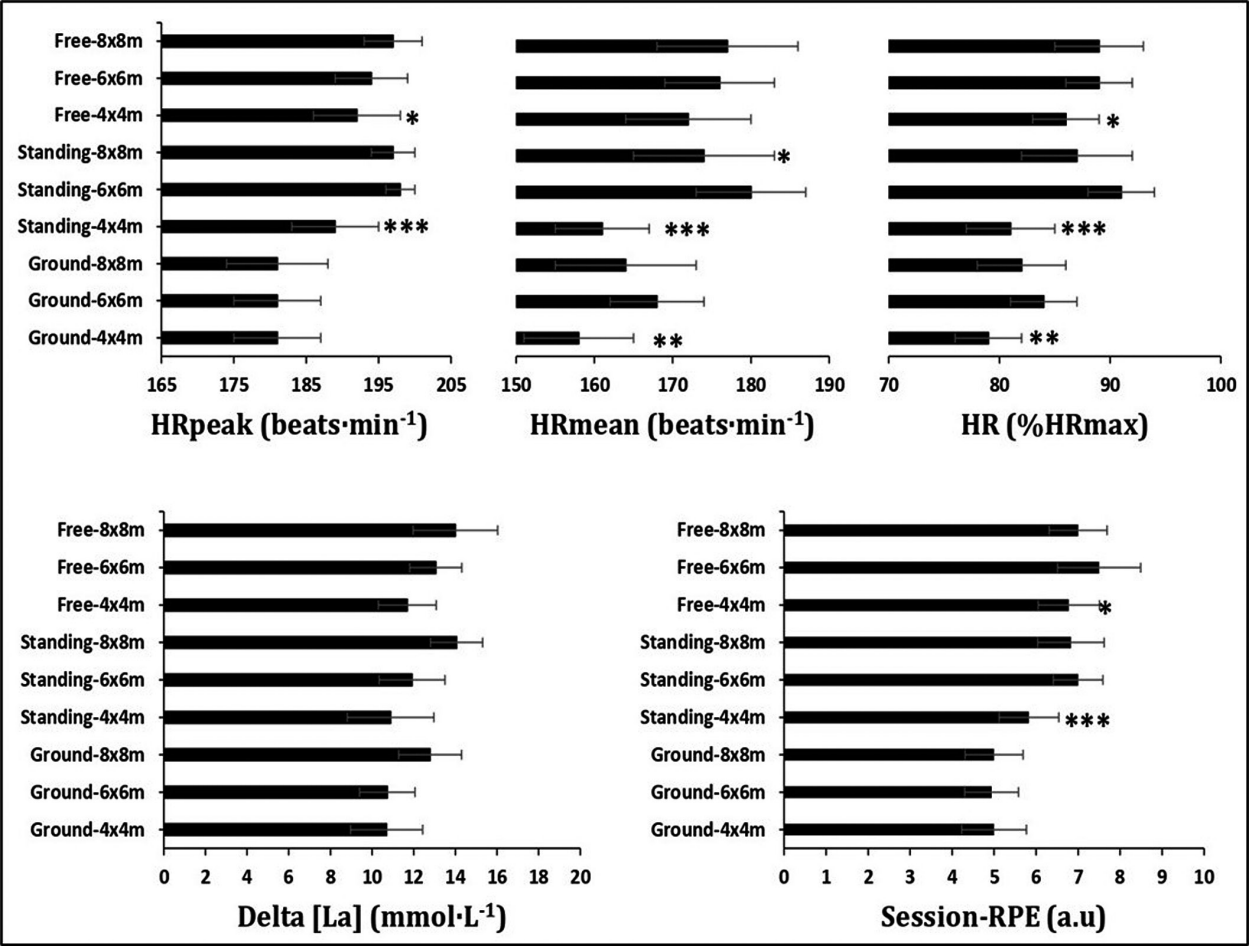
Heart rate, delta lactate concentration [La] and session rating of perceived exertion (RPE) responses during different experimental conditions resulting from the interaction between area size and training mode. * different at p < 0.05: HRmean was lower during standing combat in 8 m × 8 m compared to 6 m × 6 m; HRpeak was lower during free combat in 4 m × 4 m compared to 8 m × 8 m; %HRmax was lower during standing combat in 8 m × 8 m compared to 6 m × 6 m; Session RPE was lower during free combat in 4 m × 4 m compared to 6 m × 6 m. ** different at p < 0.01: HRmean and %HRmax were lower during groundwork in 4 m × 4 m compared to 6 m × 6 m and 8 m × 8 m. *** different at p < 0.001 HRmean, HRpeak, %HRmax and session RPE were lower during standing combat in 4 m × 4 m compared to 6 m × 6 m and 8 m × 8 m. a.u. arbitrary unit; RPE: rating of perceived exertion; HRpeak: peak heart rate; HRmean: mean heart rate; %HRmax: percentage of maximum heart rate.

When considering both area size and training mode, an interaction effect was found in HRmean (F_4,153_ = 34.048; p = 0.001), with groundwork in 4 m × 4 m eliciting lower values in comparison to 6 m × 6 m (95%CId = -16; -3; d = -1.45, large; p = 0.001) and lower values during the standing combat in 4 m × 4 m compared to 6 m × 6 m and 8 m × 8 m (95%CId = -26; -13 and -20; -7; d = -3.12 and -1.74, very large and large; p < 0.001, for all comparisons), and higher values in the standing combat in 6 m×6 m compared to 8 m×8 m (95%CId = 0;13; d = 0.79, moderate; p = 0.045). For HRpeak, there was an interaction effect (F_4,153_ = 5.493; p < 0.001), with free combat in 8 m × 8 m generating higher values in comparison to 4 m × 4 m (95%CId =1;8; d=0.86, moderate; p = 0.03) and lower values during the standing combat in 4 m × 4 m compared to 6 m × 6 m and 8 m × 8 m (95%CId = -14; -5 and -13; -4; d = -2.11 and -1.77, very large and large; p < 0.001, for all comparisons). Additionally, for %HRmax an interaction effect was detected (F_4,153_ = 5.049; p = 0.001), with higher values in 6 m × 6 m area size during groundwork compared to 4 m × 4 m in the same condition (95%CId = 2;8; d=1.47, large; p = 0.001), lower values during the standing combat in 4 m × 4 m in comparison to 6 m × 6 m and 8 m × 8 m in the same condition (95%CId = -13; -7 and -10; -4; d = -2.96 and -1.53, very large and large; p < 0.001, for all comparisons), and higher values in the standing combat in 6 m × 6 m compared to 8 m × 8 m in the same condition (95%CId = 1;6; d = 0.79, moderate; p = 0.041). Finally, an interaction effect was revealed for session RPE (F_4,153_ = 4.047; p = 0.004), with free randori in 4 m × 4 m producing lower values in comparison to 6 m × 6 m in the same condition (95%CId = -1; -0.13; d = -0.99, moderate; p = 0.012), lower values during the standing combat in 4 m × 4 m compared to 6 m × 6 m and 8 m × 8 m in the same condition (95%CId = -2; -1 and -2; -0.49; d = -1.65 and -1.42, large for both comparisons; p < 0.001, for both comparisons).

## DISCUSSION

The present study showed that 4 m × 4 m induced lower HR mean, HR peak, %HRmax, delta lactate and session-RPE values in comparison to 6 m × 6 m and 8 m × 8 m areas as well as lower values in groundwork in comparison to standing combat and free randori. Higher RPE scores were recorded in free randori compared to standing combat and lower delta lactate values in 6 m × 6 m compared to 8 m × 8 m.

Regarding the interaction between area size and training mode, the 4 m × 4 m in groundwork condition elicited lower HR mean and %HRmax values in comparison to the 6 m × 6 m in the same condition. As it was reported that male judo athletes used more immobilization, arm-locks, and choke techniques [17], the transition between these different ne-waza techniques needs larger spaces to apply than the 4 m × 4 m area, which can interrupt the transition’s continuity. However, specific time-motion analysis studies need to be conducted comparing these area sizes to check whether this explanation can be confirmed. Additionally, in the present study, lower HRmean and %HRmax values were recorded in the 4 m × 4 m area size compared to 6 m × 6 m and 8 m × 8 m in the standing combat. This result suggests that performing standing techniques in 4 m × 4 m is an irrelevant combination to stress the cardiovascular system compared to larger areas such as 6 m × 6 m and 8 m × 8 m. This may be explained by the fact that male judo athletes spend more time on performing standing techniques with high frequency of sacrifice techniques [8]. Suitable execution of such techniques requires a large space; therefore, 6 m × 6 m and 8 m × 8 m would be the ideal areas to execute these techniques. Conversely, the 4 m × 4 m area was not suitable for these techniques as it limits the offensive attempts of judo athletes via the execution of these techniques, which is confirmed by lower HR values in this area size. Furthermore, a 6 m × 6 m area in the standing condition elicited higher HR mean values (180±7 beats·min^−1^, corresponding to 91% of HRmax) than in 8 m × 8 m with the same training mode. This result was similar to what was previously reported in other investigations [18, 19, 20], suggesting, therefore, that this condition may stimulate as close as possible the competition’s physiological demands. This result can be supported by the fact that standing combat in 6 m × 6 m may provide athletes opportunities to spend more time in gripping disputes, to execute more attacks and use complex combinations [21], resulting in higher physiological demands [22]. However, the 8 m × 8 m area size may result in additional wasted time in displacement without contact [23], which may lower the physiological responses. It is relevant to note that the highest HRpeak values were recorded in the 8 m × 8 m free randori condition compared to 4 m × 4 m. Recently, it was observed that male judo athletes prefer to perform a variety of gripping [24], combined with arm technique [17] followed by groundwork attacks [24], that cannot be easily executed in reduced areas (i.e., 4 m × 4 m) but are well conducted in larger ones, such as 8 m × 8 m. It has been suggested that arm techniques result in increased cardiovascular responses compared to leg and hip techniques [5]. The findings cited above may explain our results regarding differences in HR peak responses between area sizes in free randori condition unless a technical analysis of these conditions disproves it. Thus, to obtain cardiovascular responses that mimic those observed during official judo combats (i.e., HR peak ranging from 190 to 200 beats · min^−1^ [20]), the free randori condition in 8 m × 8 m (HRpeak = 197 ± 4 beats · min^−1^) can be the most appropriate training combination. Likewise, HRpeak values recorded in the present investigation are contradictory to those previously reported by Ouergui et al. [3] in the 8 m × 8 m free randori executed by female judo athletes. These differences in physiological responses between sexes can be explained by a difference in the physical fitness and the expertise level (i.e., elite versus non-elite) [22, 25, 26] between male and female judo athletes investigated in our study and by Ouergui et al. [3].

Session RPE scores in the present study showed an interaction between conditions, while no interaction for delta lactate was found. The lowest scores recorded were in 4 m × 4 m compared to 8 m × 8 m during standing combat condition. It is well known that the gripping phase (i.e., holding the judogi) is one of the components of standing techniques [27]. Also, senior male judo athletes tend to hold the judogi for a long time [19, 28]. However, the grip duration seems shorter in the smaller area as the match is interrupted when the athletes leave the combat area, which is more likely to happen in a smaller area (4 m × 4 m) and may affect the judo athlete’s engagement and their RPE scores. Therefore, this may explain our results such as lower session RPE scores in 4 m × 4 m during standing combat. In addition, 4 m × 4 m in standing combat and free randori training modes resulted in lower session RPE values compared to when athletes performed in 6 m × 6 m area. This may be explained by the fact that the 4 m × 4 m area can result in a lower attack volume, while the larger area would be more appropriate to perform attacks without interruption. Indeed, senior middleweight male judo athletes’ (similar weight category in the present study) total combat time (233 ± 78 s) was longer compared to other weight categories, which may result in a higher attack volume [23].

It is known that judo attack phases are characterized by high-in-tensity actions stimulating the glycolytic system [4, 29, 30, 31], which may reflect the RPE scores obtained within the standing and free combat conditions in 6 m × 6 m. To highlight that, HR outcomes in the present study were higher in the 6 m × 6 m condition. Therefore, this higher physiological strain may explain the RPE scores as RPE and HR are correlated [32]. Additionally, this result may be explained by the fact that standing techniques need a larger area to be performed, especially with expert level judo athletes [21], who use a larger range of movements, such as incorporating more frequently throwing opponents forwards and backwards [33]. However, our results are not in line with those reported by Ouergui et al. [3] in female judo combat, or with striking combat sports [34, 35], which may be explained by the sex difference concerning physical fitness [22] and technical actions involved [8, 33]. Furthermore, in striking combat sports, the distance control is different from grappling combat sports, with wider distance maintained throughout the match interspersed by powerful actions (e.g., kicks, punches, elbow, or knee attacks) [34, 35], whereas in grappling combat sports, the opponents stay in close contact to execute the throwing techniques [3]. Therefore, area and training mode affect these athletes differently. The lack of analysis of technical and tactical behaviours for each condition performed is a limitation of the present study. This analysis could provide relevant information to explain the changes in physiological responses in the different experimental conditions.

## CONCLUSIONS

The study’s findings indicated that different training variables (i.e., area size of combat and type of combat) can be manipulated to differentially stress the energetic systems among male judo athletes. Namely, standing combat and free randori training modes are more appropriate to induce higher glycolytic activation and the 6 m × 6 m standing combat condition is closer to mimicking the physiological demands of the competition. In addition, the 4 m × 4 m groundwork condition could be involved in the transition phase of periodization due its lower solicitation of the glycolytic system, or for technical and tactical purposes, resulting in lower training intensity. These outcomes would be useful for judo and fitness coaches when prescribing exercise regimes, although their long-term training effects need to be investigated.
